# Developing a Chromatographic Method for Quantifying Latanoprost and Related Substances in Glaucoma Treatments

**DOI:** 10.3390/ph18050619

**Published:** 2025-04-24

**Authors:** Katarzyna Asendrych-Wicik, Katarzyna Malik, Magdalena Markowicz-Piasecka

**Affiliations:** 1 Analytical Laboratory, Research and Development Department, Pharmaceutical Works Polpharma S.A., ul. Barska 31, 02-315 Warsaw, Poland; 2 Department of Applied Pharmacy, Medical University of Łódź, ul. Muszyńskiego 1, 90-151 Łódź, Poland; magdalena.markowicz@umed.lodz.pl; 3 Research and Development Department, Pharmaceutical Works Polpharma S.A., ul. Barska 31, 02-315 Warsaw, Poland; katarzyna.malik@polpharma.com

**Keywords:** glaucoma, prostaglandin analog, latanoprost, analytical method, HPLC, benzalkonium chloride

## Abstract

**Background/Objectives**: Latanoprost is a leading active pharmaceutical ingredient belonging to the synthetic prostaglandin F2α analogs, widely used as a first-line treatment for open-angle glaucoma and increased intraocular pressure. This study addresses the critical need for an accurate and precise chromatographic method that is capable of simultaneously quantifying latanoprost and six latanoprost-related substances in antiglaucoma eye drops. This will be crucial for patient safety and treatment efficacy. This method enables the separation of latanoprost isomers, (15S)-latanoprost, latanoprost enantiomer, and 5,6-trans latanoprost from latanoprost signal. Furthermore, it is specific for the well-known latanoprost degradants—the major latanoprost acid and the minor 15-ketolatanoprost—as well as synthetic derivatives, such as triphenylphosphine oxide (TPPO) and propan-2-yl 5-(diphenylphosphoryl)pentanoate (IDPP). Using forced degradation studies using high temperatures, UV light, alkalis, acids, and oxidizing agents, the degradation profiles of the drugs were characterized and the method’s stability-indicating power was confirmed. **Methods**: Separation was achieved on a stationary combined system comprising chiral and cyano columns. Reverse-phase gradient elution and UV 210 nm detection were employed. The novel method was validated according to the European Medicines Agency International Council for Harmonisation Q2 Validation of analytical procedures—Scientific guideline. **Results**: The method was shown to be linear in the range of 40–60 µg/mL for latanoprost and 0.05–2.77 µg/mL for related substances, confirmed by a correlation coefficient of r = 0.999. Recoveries for latanoprost were obtained within the range of 98.0–102.0% for assays and 90.0–110.0% for impurities. The detection and quantification limits for latanoprost were 0.025 µg/mL and 0.35 µg/mL, respectively. **Conclusions**: The analytical procedure developed is adequately sensitive, precise, and accurate compared to existing methods. The method can be reliably used to control the critical quality attributes of low-dose latanoprost products, ensuring their required high pharmaceutical quality, which translates into improvements in patient care. This advancement holds significant implications for enhancing the therapeutic management of glaucoma, ensuring drug safety and efficacy.

## 1. Introduction

Glaucoma is a group of chronic eye disorders involving the progressive degeneration of the optic nerve and retinal nerve fiber layer (RNFL), leading to irreversible vision loss when left untreated [1]. It is a civilization disease and is the second most common cause of blindness after cataracts [2]. The World Health Organization (WHO) reports that 64 million people suffer from glaucoma worldwide [2], and the number affected is expected to increase to 111.8 million in 2040 [3]. There are several treatments for glaucoma, including topical medications, laser therapy, and surgical incision [1]. These are effective at lowering elevated intraocular pressure, which is the main disorder preceding or coexisting with glaucoma. Medical treatment offers a wide group of antiglaucoma drugs, including prostaglandin analogs (bimatoprost, latanoprost, travoprost, and tafluprost), beta-adrenergic receptor antagonists (timolol, levobunolol, metipranolol, carteolol, befunolol, and betaxolol), alpha-2 adrenergic receptor agonists (apraclonidine, brimonidine, and clonidine), carbonic anhydrase inhibitors (brinzolamide and dorzolamide), non-selective adrenergic agonists (epinephrine and dipivefrin), parasympathomimetics (pilocarpine and carbachol), osmotic drugs (glycerol, isosorbide, and mannitol), and new-generation drugs known as rho-kinase inhibitors (ripasudil and netarsudil). Of these, the first-line drugs in open-angle glaucoma and increased intraocular pressure are synthetic F2α prostaglandin analogs due to their high effectiveness, once-a-day application regimen, and good safety profiles [1]. As a result of therapy with prostaglandin analogs, an IOP reduction of approximately 30% can be achieved, similar to results obtained with the most modern therapy, i.e., selective laser trabeculoplasty (LST), which is considered a highly effective treatment.

**Figure 1:**
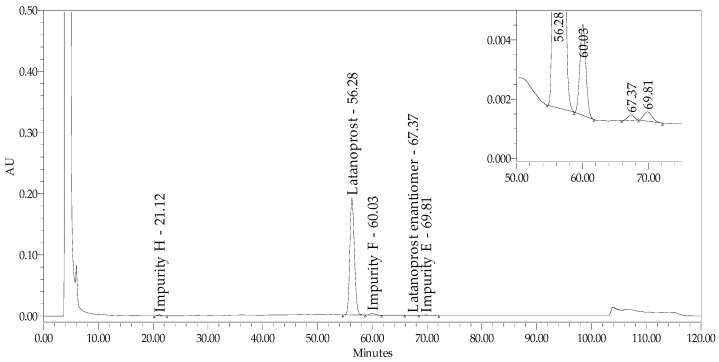
Chromatogram of test sample (preservative-free latanoprost and timolol product) spiked with impurities H (latanoprost acid), F (5,6 trans latanoprost), E (15(S)—latanoprost), and latanoprost enantiomer, demonstrating the separation achieved using stationary phase USP L93.

Latanoprost is an inactive prodrug in the form of latanoprost acid isopropyl ester. It is hydrolyzed to the active molecule latanoprost acid during corneal penetration through the action of hydrolase in the human cornea. Chemically, it is characterized as isopropyl (Z)-7-[(1R,2R,3R,5S)-3,5-dihydroxy-2-[(3R)-3-hydroxy-5-phenylpentyl]cyclopentyl]-5-heptenoate (Figure 1). The literature reports that the HPLC technique with UV detection is commonly used and suitable for determining prostaglandin analogs in both pure substances and pharmaceutical formulations [4]. Official monographs of latanoprost exist in USP-NF, and Ph. Eur.; therefore, the compendial HPLC methods with UV detection to simultaneously determine assays, as well as the ten identified organic latanoprost process impurities in APIs, are available [5,6]. These methods employ normal phase chromatography due to the highly lipophilic character of latanoprost, which is impossible to use for aqueous ophthalmic formulations. Both API monographs describe a separate method for quantifying a related substance, latanoprost acid, via RP-HPLC. An extensive literature survey revealed that apart from official pharmacopeial methods, many chromatographic methods have been developed. The majority cover only latanoprost assays in pharmaceutical formulations [7,8,9,10] or are intended for both pure substance and dosage forms [11,12]. A few more advanced HPLC methods describe the simultaneous quantification of latanoprost and secondary active substances such as netarsudil [13,14,15,16] and timolol [17,18] in the combined medicinal products. One of these methods allows us to determine latanoprost and timolol in the presence of two known impurities of latanoprost—latanoprost acid and 15(s) latanoprost—and two impurities of timolol [18]. Conversely, the other quantifies both APIs—latanoprost and timolol—and the preservative benzalkonium chloride (BAC) [17]. A chromatographic method for determining both antiglaucoma drugs—latanoprost and brimonidine—in the presence of BAC has also been published, although these active substances have not been combined into one medicinal product. Even though latanoprost has been used in glaucoma treatment for over 25 years, only one method besides pharmacopeial methods quantifies related substances in API [19], and only three can determine latanoprost impurities and degradation products in eye drop formulations [20,21,22]. One method determines latanoprost acid and 15-ketolatanoprost in a formulation with a BAC preservative using UV detection. The second employs UPLC with a tandem mass detector (MS/MS) to separate and identify oxidative degradation products of latanoprost: latanoprost acid, acid keto latanoprost (Isopropyl 7-(3,5-dihydroxy-2-(3-hydroxy-5-phenylpentyl)cyclopentyl)hept-5-oxoheptonoate, Isopropyl 7-(3,5dioxo-2-(3-oxo-5)–phenyl-pentyl)cyclopentyl)hept-5-enoate, 15-ketolatanoprost, and one unresolved structure. In 2012, a further exploration from a pending USP monograph on latanoprost ophthalmic solutions [22] was introduced for public comment. It contained an analytical method for quantifying 5,6-trans latanoprost, 15-S latanoprost and its enantiomer, and two unidentified impurities referred by their relative retention times. This is the only analytical method we have found that concerns the study of latanoprost isomers in a medicinal product. To date, there is no official USP monograph on latanoprost ophthalmic solution. In 2023, a new latanoprost USP monograph topical liquid was implemented; however, it does not contain a test for related substances.

To the best of our knowledge, none of these methods are intended for the simultaneous quantitation of latanoprost and the known latanoprost impurities (D (Propan-2-yl 5-(diphenylphosphoryl)pentanoate), H (latanoprost acid), F (5,6—trans latanoprost), E (15-S latanoprost), latanoprost enantiomer, 15-ketolatanoprost, TPPO (triphenylphospinoxide), and unidentified degradation products) in simple or complex pharmaceutical formulations, with or without the BAC preservative. Therefore, in line with ICH recommendations, this study aimed to develop and validate [23] a new reverse-phase stability-indicating chromatographic method specific to latanoprost and six related substances in eye drop solutions containing latanoprost, as well as latanoprost combined with timolol in formulations tested in the presence of benzalkonium chloride.

## 2. Results

### 2.1. Method Development

The main assumption adopted in the method development plan was to define the requirements that simultaneously determine the content and select known impurities. The following method resolution parameters (Rs ≥ 1.0) between signals due to impurities and API—symmetry factors (As) of latanoprost—peak in the range of 0.8–2.0, and the limit of quantification LOQ ≥ 0.1% is considered critical. In the pharmaceutical industry, control strategies for drug products require the monitoring of impurities originating from the degradation of active substances above a specific reporting threshold resulting from the maximum daily dose of the product used. In the case of eye drops containing latanoprost at a concentration of 0.005%, the maximum amount of latanoprost administered to the patient per day is 3 µg (1 drop (30 µL) in each eye per day). According to the ICH Topic Q 3 B (R2) Impurities in New Drug Products guidelines [24], the reporting threshold for products with a maximal daily dose ≤ 1 g is 0.1%. Therefore, all latanoprost impurities identified and unidentified in the product at a level of 0.1% must be quantitatively determined. The analytical method must be sufficiently sensitive to ensure the determination of impurities with appropriate precision at the indicated reporting level. The method used to determine latanoprost-related substances must demonstrate a quantitation limit (LOQ) of no more than the reporting threshold of 0.1%. Considering the low product concentration of 0.005% and the weak absorbance of the latanoprost molecule at 210 nm (there is only one maximum found in the latanoprost UV spectrum), an LOQ ≥ 0.1% is considered critical for the development of an analytical method. Two groups of signals were identified as being critical to achieving satisfactory separation; the first comprises latanoprost and 5,6 trans latanoprost (Impurity F), and the second comprises 15(S)—latanoprost (Impurity E) and latanoprost enantiomer. For preliminary research, a few stationary phases mentioned in the literature were screened to determine how to separate latanoprost and Impurity F. These studies involved modifying the mobile phase, gradient compositions focused on reducing organic solvent percentage, and flow changes to those described in our references (Table 1).

The results of screening studies prompted us to switch to chiral chromatography, even though it may seem that this type of technique is unnecessary for separating geometric isomers as opposed to optic ones. Chiralcel columns were used for further studies, with a mobile phase consisting of water, acetonitrile, and orthophosphoric acid or phosphate buffer with an adjusted pH of 3.0. The chromatographic conditions (Chiralcel 150 × 4.6 mm 10 µm) provided in the pending latanoprost ophthalmic solution monograph [22] were also verified, but they were found to be inappropriate. Different modifications of mobile phase composition and flow led to the expected separation of both groups of critical signals in cellulose tris (3,5-dimethylphenylcarbamate) reversed-phase chiral stationary phase coated with silica gel, but only for mono and combo products without the preservative benzalkonium chloride (Figure 1).

**Figure 2:**
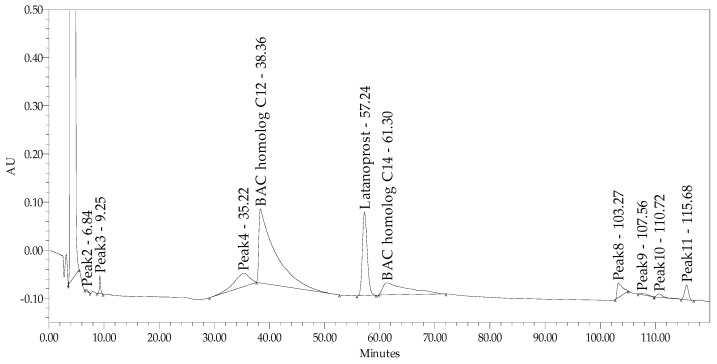
Chromatogram of test sample (latanoprost and timolol product with benzalkonium chloride (BAC) in stationary phase USP L39.

In products containing BAC, signals due to C12 and C14 homologs consumed a large chromatogram area and made it impossible to assess impurity F, impurity E, and enantiomer (Figure 2).

For this reason, a Chiralcel column with a smaller particle size of 3.5 µm was checked in the next step; however, the desired result was not obtained for products with a preservative. For preservative-free products, the sensitivity of the method was not satisfactory due to the very low peak area of impurity E and enantiomer.

**Figure 3:**
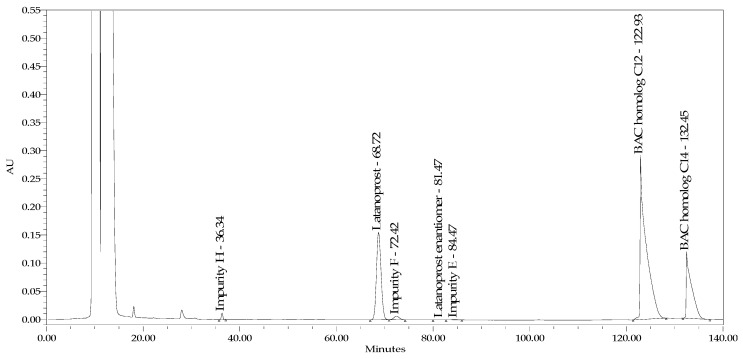
Chromatogram of test sample (latanoprost and timolol product with benzalkonium chloride (BAC) spiked with impurities H, F, E, and latanoprost enantiomer in combined stationary phase systems USP L93 and USP L10.

The main idea for the method under development was universality, enabling the tracking of specific latanoprost impurities in preparations regardless of the different excipients used. Therefore, considering the available literature on methods for determining latanoprost in preparations containing benzalkonium chloride [20], a new solution was examined. Thus, we employed a system comprising both stationary phases in a chiral and cyano column and a mobile phase consisting of water, acetonitrile, and orthophosphoric acid. The main task of the cyano column was to extend the retention of benzalkonium chloride homologs, while the chiral column was responsible for separating latanoprost isomers. The combination of stationary phases shifted the peaks of BAC C12 and C14 homologues outside the elution region of the peaks of latanoprost related substances and improved their separation, thus enabling their quantitative determination together with the content of latanoprost in the presence of a preservative. Several experiments were carried out to modify the mobile phase gradient to optimize the method and obtain the expected chromatographic parameters. Finally, appropriate separation was achieved, and the required critical parameters were obtained: Rs latanoprost/latanoprost imp F = 2.0; latanoprost enantiomer/imp E latanoprost—1.3; symmetry factor of the latanoprost peak at 100% concentration—1.5; and LOQ = 0, 1%. Figure 3 presents a chromatogram of the product containing latanoprost and timolol with BAC spiked with impurities H, F, E, and latanoprost enantiomer analyzed according to the developed method.

### 2.2. Method Validation

#### 2.2.1. System Suitability

**Figure 4:**
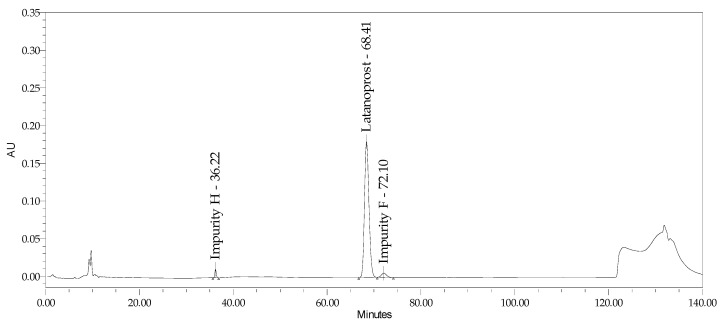
Chromatogram of system suitability testing solution.

The suitability of the chromatographic system was checked by subjecting a system suitability test (SST) solution (once) and a 1% and 100% latanoprost reference solution (five times) to Waters and Agilent systems and comparing the results against the established acceptance criteria (Table 2; Figure 4). All results met the established acceptance criteria.

#### 2.2.2. Specificity and Stability-Indicating Power

**Figure 5:**
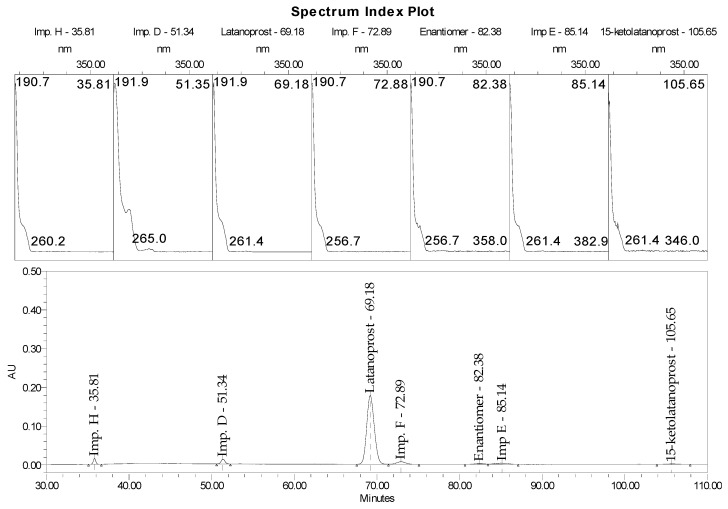
Chromatogram of test sample (latanoprost and timolol product with benzalkonium chloride) spiked with impurities H, D, F, E, 15-ketolatanoprost, and latanoprost enantiomer.

**Figure 6:**
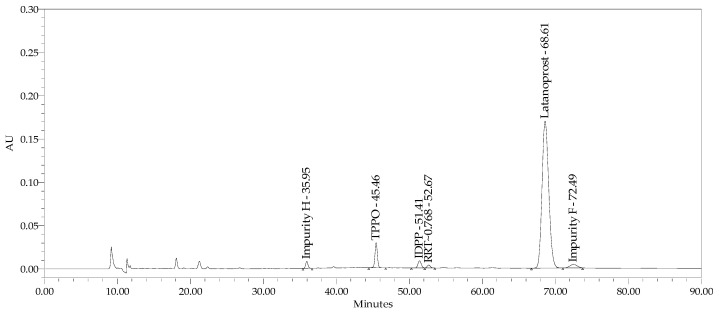
Chromatogram of Xalatan spiked with impurity D (IDPP) and TPPO at ~1.0%.

The specificity of the method was examined by comparing the retention time and UV spectrum in the range of 190–400 nm obtained for the following reference solutions: impurities D, E, F, H, latanoprost enantiomer, and 15-ketolatanoprost with parameters obtained for product samples; products spiked with latanoprost impurity H, latanoprost impurity F, latanoprost impurity E, and 15-ketolatanoprost at levels of ca. 2.0%, 3.5%, and 0.5%; and latanoprost enantiomer at about 0.15% in relation to the declared latanoprost content in eye drops (Figure 5). We also examined the method specificity for TPPO, as described in other studies. In parallel, TPPO standard solution at a concentration of 1% and Xalatan product spiked with 1% TPPO were analyzed. A chromatogram of the Xalatan product with added impurities (TPPO and IDPP) is presented in Figure 6. The chromatograms (Figure 5 and Figure 6) show that all peaks originating from known impurities are well separated from other peaks, as well as from the active substance signal.

To confirm the stability-indicating power of the tested method and to investigate the main potential degradation products of latanoprost, forced degradation studies under severe physical and chemical conditions were performed. Degrading agents were selected such that the active substance was reduced to a maximum of 30%. In the case of alkaline hydrolysis, using 0.2 M NaOH led to the complete hydrolysis of the ester and the formation of 100% latanoprost acid. Therefore, milder conditions were used, i.e., half the concentration of NaOH for the decomposition of latanoprost, and only the product without timolol showed an increase in degradants. The mass balance for all reaction types was within a range of 95–105%, indicating the suitability of the method for monitoring the stability of both mono and combo product formulations. The results are presented in Table 3. The chromatograms obtained during the degradation studies are included in the Supplementary Materials Section.

The forced degradation studies indicated that two known latanoprost process impurities, H and F, originating from organic synthesis (listed in latanoprost Ph. Eur. monograph 2230) may form in drug products as a result of degradation. Impurity H can be generated from different types of degradation, both through alkaline and acidic hydrolysis, as well as under elevated temperatures. Since ophthalmic products are aqueous solutions that promote hydrolysis reactions, impurity H should be considered the main and most typical degradation product. Chemical impurity H is free latanoprost acid formed through the hydrolysis of inactive latanoprost prodrug in the form of isopropyl ester into a pharmacologically active moiety. The literature thoroughly and commonly notes this as the main degradant of aqueous latanoprost solutions [20,21]. In vivo, it forms very rapidly when latanoprost is hydrolyzed by esterase enzymes, which are abundant in the cornea [26]. Our forced degradation studies showed differences between the degradation pathways of the mono and combo products. During a review of the data, we found that the product with timolol was more susceptible to UV light. After one hour of exposure to UV light, the latanoprost content in the combined product decreased to 70%; at the same time, impurity F increased by 22%, while the mono product remained unchanged compared with the unexposed product. There are no references to the degradative nature of impurity F in the literature. The pending USP monograph [22] indicates the need to assess impurity F content in products, but this requirement may be associated with a USP monograph limit for pure substances, amounting to 3.5%. This exceeds identification and qualification levels according to the ICH guidelines and, thus, requires traceability in products. Oxidizing the C-15 hydroxyl group without isopropyl ester hydrolysis produces 15-ketolatanoprost [20]. Although the literature mentions this as a minor degradant of latanoprost in tested mono and combo products with BAC, it does not appear after degradation. It is reported that aqueous solutions of latanoprost are unstable and highly sensitive to light and increased temperatures, thus forming degradation products, namely, latanoprost acid and 15-ketolatanoprost [20,21]. Notably, for the mono products with or without BAC, the effect of light was not significant; however, for the combination products, there was a high degree of latanoprost degradation. A significant increase in impurity F was observed, probably related to timolol in the drug composition. Further research is needed to explain and understand the reaction mechanisms.

#### 2.2.3. System Precision

The measuring system’s precision was assessed by applying six injections of latanoprost standard solutions to the chromatographic column at concentrations of about 50 µg/mL (100% in relation to the declared latanoprost assay in the medicinal product) and 0.50 µg/mL (~1.0% in relation to the declared latanoprost assay in the medicinal product), along with a model sample of the product with timolol and BAC, spiked with three latanoprost impurities (H, F, and E) at a ~1.0%, ~1.8% and ~0.3% level, respectively (which correspond to actual concentrations of 0.50 µg/mL, 0.88 µg/mL, and 0.14 µg/mL, respectively). The variability in peak areas and retention times obtained from six injections was evaluated. The results are presented in Table 4.

#### 2.2.4. Method Precision, Linearity, and Accuracy

The repeatability of the method was determined by analyzing six independent samples of products spiked with impurities H, F, and E. To investigate the intermediate precision of the test method, the following changes to the measurement conditions were made: a different analyst; a different day of analysis; a different HPLC system; and different reagents. The results were statistically analyzed; to determine the confidence interval of the mean value, we estimated statistical parameters such as the mean, SD, and RSD% for six and twelve samples. The linearity of the assay for latanoprost-related substances in the tested product was verified by assessing five concentration levels of impurity H for a range of 0.10% (RT) ÷ 3.5%, impurity F for a range of 0.10% (RT) ÷ 5.5%, impurity E for a range of 0.10% (RT) ÷ 0.8%, and latanoprost for a range of 0.10% (RT) ÷ 5.5%. For each concentration, level 3 model samples were prepared containing all product excipients and known quantities of the substance being determined.

**Figure 7:**
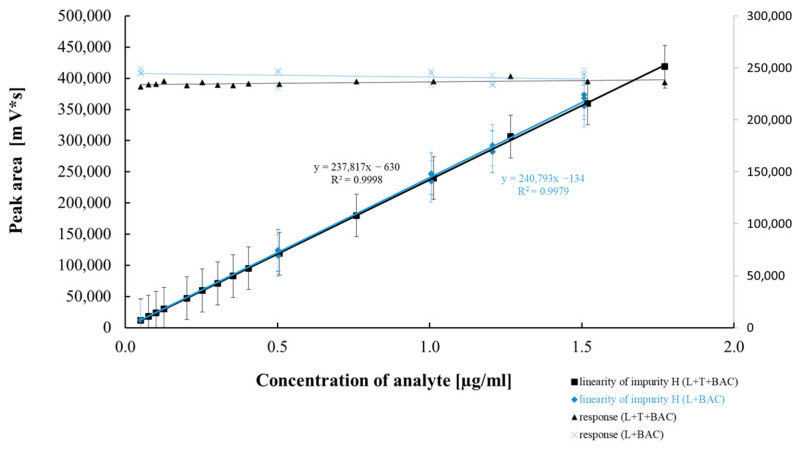
Linear correlation between impurity H concentration and peak area.

**Figure 8:**
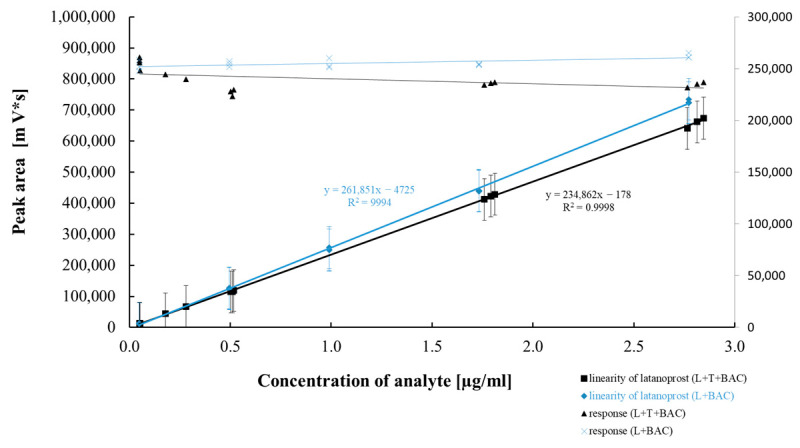
Linear correlation between latanoprost concentration and peak area.

**Figure 9:**
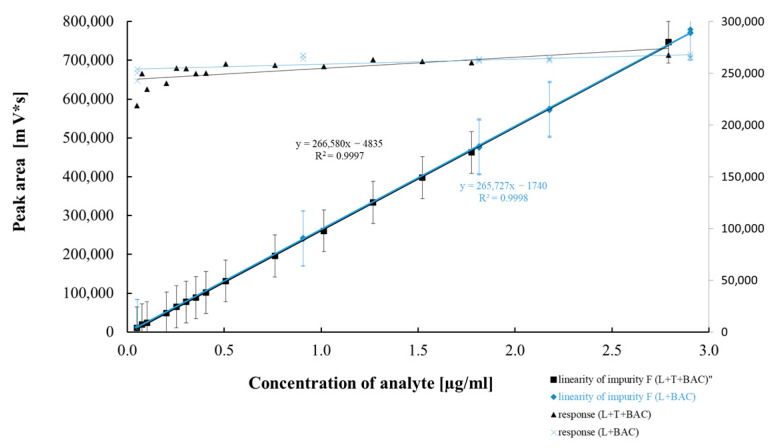
Linear correlation between impurity F concentration and peak area.

**Figure 10:**
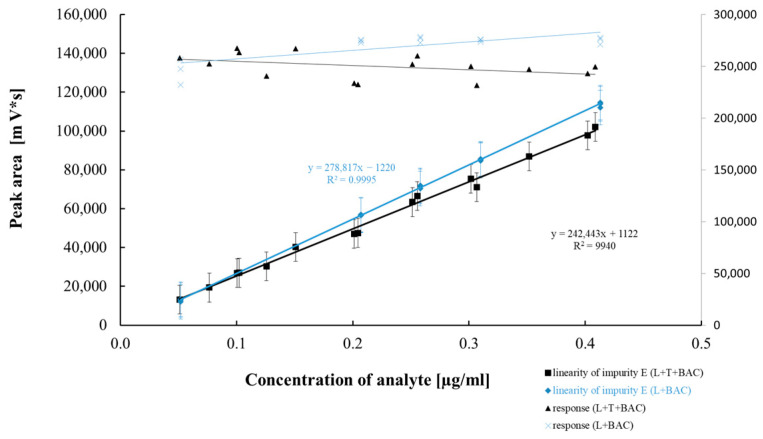
Linear correlation between impurity E concentration and peak area.

Table 5 and Table 6 summarize the results of the statistical assessment of the method’s analytical precision, linearity, and accuracy for both mono and combo products with the preservative BAC. Figure 7, Figure 8, Figure 9 and Figure 10 present regression curves for latanoprost and the tested specified impurities obtained via the least squares method.

A positive linear correlation was confirmed between the analyte concentration and the signal measured for latanoprost and the tested impurities (E, H, and F). This was evidenced by correlation coefficients very close to 1.0 and the *p*-values (Table 5 and Table 6) calculated for the calibration curves, all of which were significantly below the assumed confidence level (*p* = 0.05) and confirmed that the linear model correlation is statistically significant.

The analytical method repeatability and intermediate precision studies showed that the method met the assumed acceptance criteria in terms of the RSD values obtained for six repetitions, as well as for the intermediate precision study of twelve repetitions covering random changes that may affect the values determined. The latanoprost assay RSD% values in the precision studies (repeatability and intermediate precision) for both tested products were below the predicted RSD values of 2.0% and 4.0% for repeatability and intermediate precision, respectively. Based on the AOAC Official Methods of Analysis (2016) Guidelines for Standard Method Performance Requirements Appendix F, we adopted the expected acceptance criteria for the precision study of the method regarding related substances [27]. According to these guidelines, expected precision is a function of analyte concentration. Consequently, for the analyte present at a level of 0.1 µg/mL (0.1 ppm), i.e., impurities H, E, and latanoprost (at a concentration for the evaluation of the unknown impurity), the expected RSD values for the repeatability and intermediate precision study are 15% and 22%, respectively; conversely, for impurity F—tested at a concentration of approximately 1 ug/mL (1 ppm)—they are 11% and 16%, respectively. All results were significantly lower than the assumed critical values, and the highest RSD value (*n* = 12) of 18.1% was reported for impurity E in the intermediate precision study of the latanoprost mono product. Considering the variability at such a low concentration of impurity E (0.11 ug/mL), it should be pointed out that the difference in the average absolute impurity E contents between the tests—performed on different days by a different analyst using different reagent lots and chromatographic systems—equaled only 0.05 ppm (ug/mL). This demonstrates the good precision of the tested method. In addition, the data from the linearity studies, presented in Figure 7, Figure 8, Figure 9 and Figure 10, were also assessed in the context of method reproducibility. In all cases, 15 measurement points of the tested concentration range were within the assumed precision of the method. Table 7 presents the RSD(*u*) values obtained from the method linearity test for 15 model samples for each impurity and latanoprost. The *u* value is the response factor calculated according to the following formula:

$$u=\frac{P}{C}$$

where:

*P*—peak area of the analyte in the model sample [µV × s];
*C*—concentration of the analyte in the measured model sample [µg/mL].

**Table 7:** Precision of response factors from linearity studies.

	Latanoprost Assay	Unknown Impurity	Impurity H	Impurity E	Impurity F
RSD (*u*) *n* = 15 L + T + BAC	0.74%	4.7%	1.1%	4.8%	5.0%
RSD (*u*) *n* = 15 L + BAC	0.44%	2.0%	2.5%	4.7%	2.6%

In summary, the precision of the method was suitable for identifying latanoprost and its related substances in low-dose medicinal products.

#### 2.2.5. Limit of Detection and Quantification

According to guideline ICH Q2 (R2) for the determination of the limits of detection (LOD) and quantification (LOQ), several approaches can be used: visual evaluation based on calibration curves, or via s/n parameter assessment. The LOD and LOQ were estimated using the standard deviation of a linear response and a slope according to formulas *DL* and *QL*, respectively, presented below:

$$DL=\frac{3.3\times \sigma }{S}$$

$$QL=\frac{10\times \sigma }{10}$$

where σ is the standard deviation of the response, and *S* is the slope of the calibration curve.

The calculated values were then experimentally confirmed based on a latanoprost solution at a concentration close to these values. Table 8 presents the LOD and LOQ values calculated based on the regression curves for latanoprost and impurities F, H, and E tested in both mono and combo products.

In the next step, the estimated limit values were experimentally verified through a six-fold analysis of latanoprost solutions at concentrations of 0.025 µg/mL, 0.035 µg/mL, and 0.050 µg/mL. The signal-to-noise ratio and standard deviation of the signal areas from six injections were determined. The results were evaluated against the following requirements: for LOD, a signal-to-noise ratio of no less than 3; for LOQ, a signal-to-noise ratio of no less than 10; and for RSD, an *n* = 6 of no more than 5.0%. Table 9 presents the experimentally determined limits.

The experimentally determined limit of quantification was less than the adopted reporting threshold value of 0.050 µg/mL (0.1% in relation to the declared latanoprost content in the tested products), indicating its adequate high sensitivity for determining the purity of low-dose products.

#### 2.2.6. Robustness

The robustness of an analytical procedure is a measure of its capacity to remain unaffected by small but deliberate variations in method parameters. This indicates its reliability during normal usage [23]. The effects of small changes in relation to those specified in the method were verified by conducting an analysis under different experimental conditions. The following factors were considered: changes in mobile phase composition (±2% acetonitrile); changes in column temperature (±2 °C); and changes in the chromatographic set (apparatus and column serial numbers; detector types: UV and PDA). To determine the method’s robustness, a stability study was performed on the test samples, latanoprost reference solutions and the SST solution under standard solution storage in an autosampler chamber and a refrigerator. We compared SST parameters from the chromatograms of the reference solution and the SST solution obtained under different test conditions. In all analyzed cases, the required SST conditions for the analytical method were met (Table 10). A minor change in the analysis conditions did not affect the results of the latanoprost assay, and the RSD% values for all change types ranged within the expected method precision, i.e., RSD ≤ 2.0% (Table 11). In the case of testing related substances, the impurity results under different testing conditions varied within the precision of the method. However, the range of changes in acetonitrile (+/−2%) in relation to those set in the procedure was not exceeded due to the high RSD value for the determined impurity E content (Table 11). In conclusion, the accuracy of preparing the mobile phase should be strictly observed within the limits established during the method’s robustness test.

Stability studies confirmed that, when stored in a refrigerator (temperature, 5 °C ± 3 °C), the standard latanoprost solution was stable for 10 days, and the SST solution was stable for 3 months.

### 2.3. Drug Product Analyses

**Figure 11:**
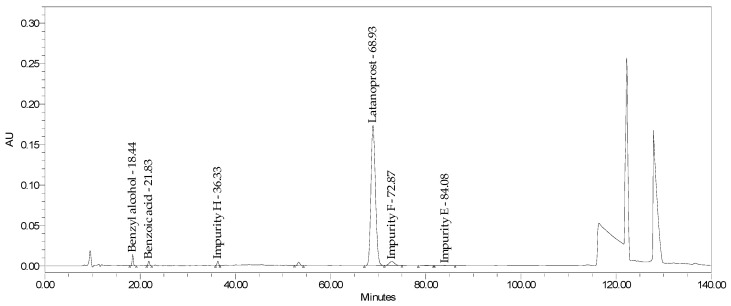
Chromatogram of Xalatan.

**Figure 12:**
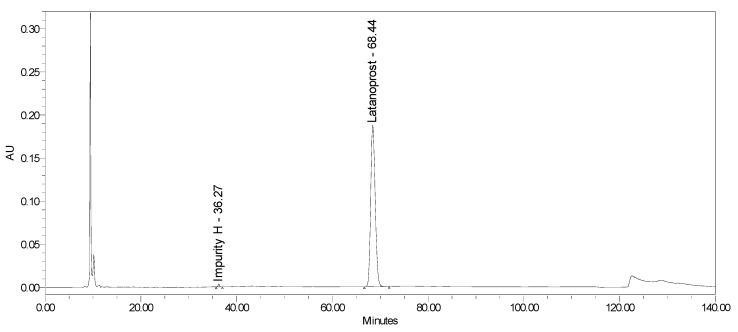
Chromatogram of benzalkonium chloride-free latanoprost formulation.

This study aimed to develop a method of simultaneously identifying latanoprost content and its six impurities (including geometric and optic isomers) in eye drops containing latanoprost with or without benzalkonium chloride. This method was validated based on the formulations of Xalacom and Xalatan products. It was also verified for generic products without the preservatives latanoprost and latanoprost with timolol. The results indicate the possibility of applying this procedure to different low-dose latanoprost pharmaceutical formulations. Summaries of the analytical results and chromatograms obtained for the Xalacom, Xalatan, and generic BAC-free products are presented in Table 12 and Table 13 and Figure 11 and Figure 12.

## 3. Discussion

To ensure the high quality and safety of a medicinal product, it is extremely important to develop methods that can characterize impurity profiles specific to that pharmaceutical formulation. Latanoprost is a very potent substance that is relatively susceptible to decomposition under the influence of temperature, light, oxidizing agents, and hydrolytic factors. As a result of degradation, known structural impurities and unidentified degradation products are formed. Considering the structure of the latanoprost molecule—characterized by the presence of five chiral carbons and several double bonds—the number of potential degradation and transformation products (including isomers) is very high, and their biological activity is not known. Some latanoprost impurities show biological activity. Impurity H (latanoprost acid) is the metabolite of latanoprost, with 200-fold higher activity at the prostaglandin F2α receptor than latanoprost [4]. Similarly, 15-ketolatanoprost is an active metabolite; at a concentration of 0.001%, it lowers pressure in glaucomatous monkey eyes, equivalent to the effect of 0.005% latanoprost [28]. Therefore, it is very likely that other identified and unidentified impurities of latanoprost also exhibit biological activity; however, their strengths and directions are unknown. Thus, to ensure the safe and targeted therapeutic effects of drugs containing latanoprost, it is essential to strictly control known and unknown impurities and keep them at the lowest possible level.

The developed and validated method controls the active substance’s content, six known latanoprost impurities, and the enantiomeric purity of the product. This seems to be particularly valuable during development research when it is necessary to collect the widest possible knowledge regarding degradation products, their formation paths, and the stability of their formulations.

The advantage of this method is that it can test mono or combination products. It can identify differences between the tested compositions in terms of the impurity profile and stability of the formulation. Indeed, it was possible to demonstrate that impurity F is characteristic of complex formulations and may not merely be due to API synthesis, as described in the literature. Using this test method to analyze contents and related substances and assess enantiomeric purity for two products translates into economic profit due to a reduction in the standards used and materials necessary to conduct the analysis. Moreover, using in situ degradation to generate impurity H in the preparation of an SST solution also has an economic aspect; this avoids the need to purchase expensive standards (for example, Latanoprost Impurity H Reference Standard costs EUR 1000 for 0.0.002 mg). Our method is accurate, linear, and precise, and it is suitable for its intended purpose. It should be emphasized that it is sensitive, which is crucial for low-dose pharmaceutical products for which the reporting threshold is set to 0.1% based on the dose recommended by the ICH [24]. The weakness of the methodology is an analysis duration of 140 min; however, considering the number of data that can be gained from one analysis, it seems that the benefits outweigh the time consumption.

The use of this method in other excipient compositions seems possible, but requires further research to verify the selected validation characteristics. The developed analytical method was tested with low-dose latanoprost products with known compositions: Xalacom, Xalatan, and generic products without preservatives. These products differed in excipients. Therefore, for each composition, the specificity of the method was confirmed by proving the following: the peaks originating from the components of the vehicle and its degradation and, in the case of the complex product, the peaks originating from the second active substance (i.e., timolol and its impurities) do not interfere and are adequately separated (Rs ≥ 1.0) from the latanoprost peak, the signals originating from its specified impurities, and its unknown degradation products. When using this method for other low-dose latanoprost products with complex excipients or a second API, it would be necessary to perform specificity studies, with a particular emphasis on degradation studies conducted separately for the vehicle/matrix and the product to identify signals originating from excipients and their possible degradation products. It would also be necessary to assess their impact on the possibility of correctly identifying latanoprost and its related substances. Considering the low ratio of the latanoprost concentration to the concentrations of the excipients used or the second API (for example, the timolol concentration is 100 times higher than the latanoprost concentration, and the amount of netarsudil in the complex product with latanoprost is 4 times higher than in latanoprost) and the high sensitivity of the method necessary to assess latanoprost-related substances at a reporting level above 0.1% in relation to the declared content of 50 ug/mL; the main limitation of the method is that the signals from the excipients or second API (as well as their degradation products) are visible on the product chromatograms and may make it difficult to distinguish peaks from latanoprost impurities. In addition, the low concentration and weak absorbance of latanoprost cause peaks in the vehicle/matrix, and its degradation can be significantly higher than the peaks of latanoprost impurities. This may translate into difficulties in correctly identifying peaks originating from unknown latanoprost impurities and the vehicle in routine analysis. An example of the above-mentioned issue in Xalacom and Xalatan analyses is benzyl alcohol, the main BAC degradant, which is visible on chromatograms at a signal of RT ~18.4. Peak assessment at the degradation study stage is based on the UV spectrum; reference standard solutions can identify this peak and exclude it from the assessment. However, incorrectly treating this signal as a latanoprost degradation product would provide false results in the product quality assessment and the OOS results in terms of impurities. Similarly, a timolol degradation product was also detected in the chromatogram of combo products after degradation. Therefore, gaining in-depth knowledge of the product during the forced degradation of individual product components and using available reference standards and additional support with UV spectra are essential for correctly evaluating chromatograms. One of the proposed solutions is introducing a vehicle/matrix test as a blank in standard analysis of performance, which can significantly facilitate the assessment of chromatograms and peak identification. Nevertheless, given the demonstrated specificity of this method, it can be successfully used as a platform method for other low-dose products containing latanoprost, even those with complex excipients or a second API, such as timolol or netarsudil.

The main purpose of this method is as follows: It monitors the degradation products of latanoprost in various formulations at the development stage to (1) show all of the potential impurities that may appear because of aging throughout its shelf life and (2) to highlight the significant effect of its composition on any impurities that form. Therefore, the method was developed to monitor as many impurities as possible. The original purpose of the method clearly does not exclude the possibility of its use for routine control analyses, as was confirmed by our validation.

A minimal method development approach was used based on the ICHQ14 definition [29]. Robustness studies of the method were carried out during validation through the univariate examination of a single parameter. Considering the available optimization tools in the enhanced approach to method development, it is interesting to optimize the method using multivariate experiments (DoEs) to establish the multifactorial dependencies of the changed parameters. This provides a better understanding of the relationships between the variables of the analytical procedure variables and its responses. Due to the complex system of stationary phases and the length of the analysis, in confirmed cases after determining potential degradation products in forced degradation studies and long-term stability studies, this method could be individually adapted to monitor only the selected impurities that are likely to occur in the commercial batches of a medicinal product. This approach would significantly reduce analysis time, leading to its greater usability and ease of use in routine product quality control. Considering the method’s potential, it can also be optimized to simultaneously identify latanoprost, its impurities, and benzalkonium chloride degradants such as benzyl alcohol and benzoic acid. These have high and symmetrical signals, potentially useful for quantitative determination (Figure 11).

## 4. Materials and Methods

### 4.1. Materials

**Figure 13:**
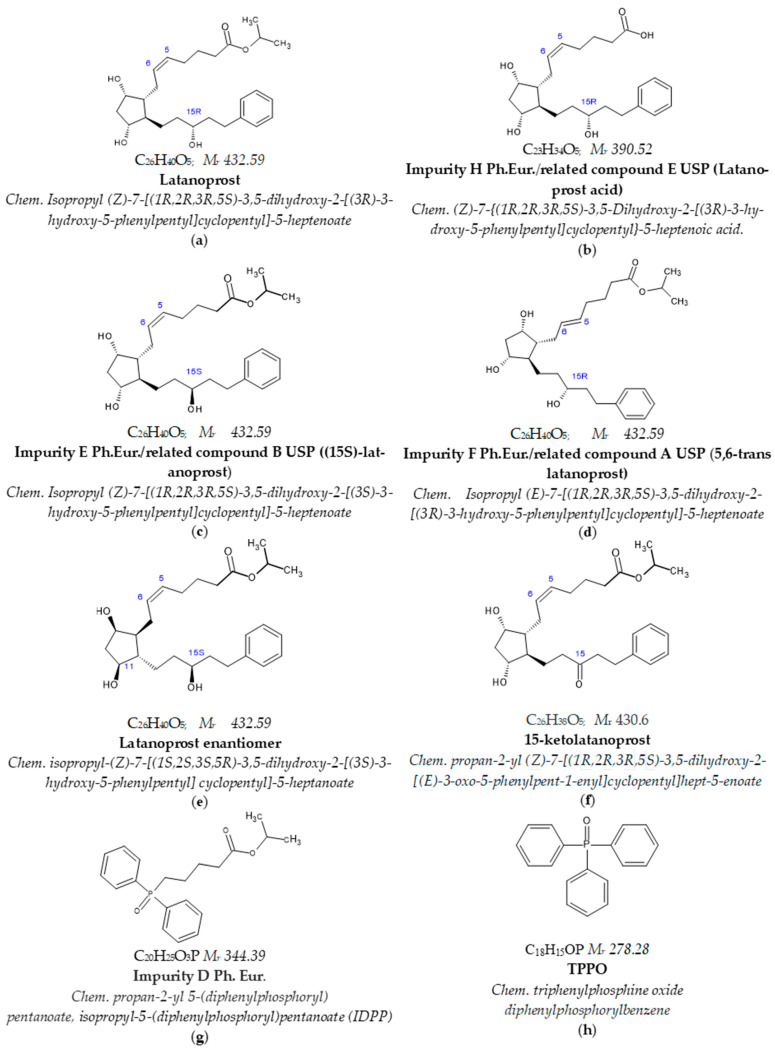
Chemical structures of latanoprost and tested related substances. (**a**) Latanoprost, (**b**) latanoprost acid, (**c**) (15S)-latanoprost, (**d**) 5,6-trans latanoprost, (**e**) latanoprost enantiomer, (**f**) 15-ketolatanoprost, (**g**) IDPP, and (**h**) TPPO.

Latanoprost API, latanoprost working standard, the reference standards of latanoprost impurities H, F, and E (according to Ph. Eur. nomenclature), and latanoprost enantiomer were obtained from YS Life Science Ltd., previously Yonsung Fine Chemicals co., LTD (Jangan-myeon, Republic of Korea). The reference standard of impurity D (according to Ph. Eur. nomenclature) was purchased from Toronto Research Chemical (Toronto, ON, Canada), and TPPO was purchased from Sigma-Aldrich (Burlington, NJ, USA). Figure 13 presents the structures with chemical and compendial Ph. Eur. and USP names with corresponding common names for latanoprost and the tested impurities.

Acetonitrile, the gradient grade for HPLC, was purchased from VWR Chemicals (Radnor, PA, USA). 85% orthophosphoric acid, HPLC-grade purity, and sodium phosphate monobasic (assay ≥ 99.0%) for HPLC were manufactured by Fluka (Buchs, Switzerland). Pure sodium hydroxide and 35–38% hydrochloric acid (pure for analysis) were purchased from POCH Poland (Avantor Performance Materials Poland S.A., Gliwice, Poland). Pure sodium chloride and 30% pure hydrogen peroxide were purchased for analysis from Merck (Darmstadt, Germany). Water for HPLC was obtained via the Milli-Q water purification system Advantage A10 Millipore (Merck KGaA, Darmstadt, Germany).

The tested samples, Xalatan, 50 µg/mL, and Xalacom, (50 µg + 5 mg)/mL, were purchased from the EU market. To develop and validate the analytical methods, model products corresponding to the qualitative and quantitative composition of the original formulations were internally prepared. In addition, the new preservative-free generic formulations containing latanoprost (50 µg/mL) and latanoprost with timolol ((50 µg + 5 mg)/mL) were examined. Latanoprost standard stock solution in acetonitrile was used to prepare the reference solution at concentrations of 50 µg/mL and 0.5 µg/mL via dilution with phosphoric buffer adjusted to pH 6. The solution for chromatographic system suitability testing (SST) contained latanoprost and known major latanoprost impurities F and H at levels of about 3.5% and 2.0%, respectively. In the SST solution, impurity H was generated in situ via acid hydrolysis of latanoprost, and impurity F was generated by spiking the latanoprost stock solution.

### 4.2. HPLC Analysis

Two HPLC systems were used: an Agilent (Santa Clara, CA, USA) HPLC system equipped with a UV detector and a Waters (Milford, MA, USA) HPLC system connected to a photodiode array detector (PDA). Both systems were controlled by the Empower 3 software (Waters, Milford, MA, USA). The stationary phase system consisted of nitrile groups chemically bonded to porous silica particles (USPL10) and cellulose tris (3,5-dimethylphenylcarbamate) reversed-phase chiral stationary phase coated on silica gel (USP L93). Two mobile phases were used in the HPLC system. Mobile phase A, a mixture of acetonitrile, water, and 85% orthophosphoric acid (300:700:5), and mobile phase B, consisting of 100% pure acetonitrile, were pumped in gradient mode (Table 14). Undiluted products were applied to the chromatographic column. Data were collected in the range of 190–400 nm.

### 4.3. Method Validation

The method validation was validated according to ICH Q2 Validation of Analytical Procedures: Methodology [23]. The following performance characteristics were verified: specificity, system precision, repeatability, intermediate precision, linearity, accuracy, limit of detection, limit of quantification, and robustness.

Forced degradation studies were conducted on mono and combo model products with BAC and the matrix of a combo product containing one API, i.e., latanoprost or timolol maleate and vehiculum. Drug products, vehicula, and matrixes were treated with 30% H_2_O_2_, 5 M HCl, and 1 M NaOH (concentrations of degrading agents in a mixture of 3% H_2_O_2_, 0.2 M NaOH, and 0.2 M HCL). The hydrolyzed acid and base samples were neutralized after 4 h of exposure using 0.2 M HCL and 0.2 M NaOH, respectively. For the photodegradation study, the samples were exposed for 1 h using a 450 W/m^2^ xenon lamp as the light source, with a combined C-band filter. Elevated temperature studies were conducted at 80 °C for 24 h in a glass flask. All samples, i.e., model, mono, and combo products with BAC, vehicula, and 100% latanoprost standard solution, were subjected to forced degradation studies using the same degrading agents presented above. Assays and impurity contents were assessed in relation to standard solutions and then mass balance (Mb) was calculated according to the formula presented below:

$$Mb=\frac{Ad+Id}{A+I}\times 100\%$$

where:

Mb—mass balance [%];
A—assay of the analyte before degradation;
Ad—assay of the analyte after degradation;
I—sum of impurities before degradation;
Id—sum of impurities after degradation.

## 5. Conclusions

A new sensitive, accurate, and precise chromatographic method has been developed for the simultaneous quantification of latanoprost and six latanoprost-related substances in low-dose antiglaucoma eye drops containing latanoprost at a concentration of 50 µg/mL. This method separates latanoprost isomers, (15S)-latanoprost, latanoprost enantiomer, and 5,6-trans latanoprost from latanoprost signals. Furthermore, it is specific to the well-known latanoprost degradants—the major latanoprost acid and the minor 15-ketolatanoprost—as well as the synthetic derivatives—triphenylphosphine oxide (TPPO) and propan-2-yl 5-(diphenylphosphoryl)pentanoate (IDPP). It is suitable for testing simple and combined latanoprost products with timolol maleate (5 mg/mL) preserved with benzalkonium chloride or without preservatives. This method is a valuable tool in control strategies for low-dose latanoprost products, ensuring the safety of their use and their targeted therapeutic effects. Furthermore, this method is a potential platform for testing formulations of various complexity containing 0.005% latanoprost.

## Figures and Tables

**Table 1:** Verification of available analytical methods for resolution of imp. F and latanoprost.

Stationary Phase	Column Characteristic		Mobile Phase	Latanoprost RT [min]	Impurity F RT [min]	Resolution	Reference
	Dimension [mm]	Particle Size [µm]					
Symmetry C18	150 × 4.6	5	Phase A: 1.36 g/L KH_2_PO_4_ pH 6.8: ACN (700:300) Phase B: ACN: MeOH 1:1 (*v/v*)	40.2	40.4	Not separated	[13]
Intersil ODS	250 × 4.6	5	Phase A: 0.1 M KH_2_PO_4_; Phase B: ACN A:B (50:50 *v/v*)	469.4	501.7	Separated *	[15]
Spherisorb Phenyl	150 × 4.6	5	Octanosulphonic acid sodium salt 2.5 g/L of pH 2.5: ACN (70:30 *v/v*)	13.2	13.0	Not separated	[25]
Hypersil BDS CN	250 × 4.6	5	Phase A: 0.05 M sodium phosphate Na_3_PO_4_; pH 3.2; Phase B: ACN: MeOH (1:1 *v/v*), A:B (70:30 *v/v*)	18.4	17.9	Not separated	[20]

* Separated only when 100% of A phase is used, but where a very, very long time of retention (RT ~500 min) is not acceptable to standard application. The addition of phase B, i.e., acetonitrile, resulted in coelution.

**Table 2:** System suitability test with Agilent and Waters HPLC systems.

Results Agilent 1210 System UV/VIS Detector	Results Waters System/PDA Detector	Acceptance Criteria
Rs_latanoprost/impF_ = 2.0	Rs_latanoprost/impF_ = 2.0	Rs_latanoprost/impF_ ≥ 1.8
Std_1%_ RSD (%)_n=5_ = 1.0	Std_1%_ RSD (%)_n=5_ = 3.6	Std_1%_ RSD (%) ≤ 5.0
Std_100%_ RSD (%)_n=5_ = 0.3	Std_100%_ RSD (%)_n=5_ = 0.4	Std_100%_ RSD (%) ≤ 2.0
Std_1%_ As = 1.0	Std_1%_ As = 1.0	0.8 ≤ As ≤ 1.5
Std_100%_ As = 1.0	Std_100%_ As = 1.0	0.8 ≤ As ≤ 1.5

**Table 3:** Percentage summary of related substances and a latanoprost assay with an evaluation of the spectral peak purity of latanoprost and mass balance after forced degradation studies of model mono and combo products with benzalkonium chloride preservative.

Type of Degradation/ Product	Identified Latanoprost Impurities Content ^1^	Other single Impurities Content % ^1^	Sum of Impurities	Latanoprost % Content ^1^	PA/PT ^3^ Latanoprost	Mass Balance
Oxidation 3% H_2_O_2_ by 5 h						
L+T+BAC	Imp. H—0.21%	RRT 0.65—0.87% RRT 0.66—0.72%	1.8%	92.7%	0.394	95.0%
L+BAC	Imp. H < RT ^2^	RRT 0.65—0.17%	0.2%	94.0%	0.558	94.7%
Photolysis UV light 1 h						
L+T+BAC	Imp. F—22.9% Imp. H—0.75%	RRT 0.29—0.11% RRT 0.55—0.14% RRT 0.57—0.21%	24.1%	70.8%	0.414	95.4%
L+BAC	Imp. F < RT ^2^ Imp. H < RT ^2^	<R T^2^	NA	98.7%	0.471	99.2%
Alkaline hydrolysis 0.1 M NaOH by 4 h						
L+T+BAC	Imp. H < RT ^2^	<RT ^2^	NA	96.9%	0.295	97.4%
L+BAC	Imp. H—3.30%	<RT ^2^	3.3%	93.0%	0.299	96.8%
Alkaline hydrolysis 0.2 M NaOH by 4 h						
L+T+BAC	Imp. H—99.7%	<RT ^2^	99.7%	0.3%	0.275	100.5%
L+BAC	Imp. H—99.7%	<RT ^2^	99.7%	0.2%	0.562	100.4%
Acid hydrolysis 0.2 M HCl by 2 */4 ** h						
** L+T+BAC	Imp. H—0.41%	<RT ^2^	0.41%	93.2%	0.570	97.3%
* L+BAC	Imp. H < RT^2^	<RT ^2^	NA	98.3%	0.483	98.2%
Thermal degradation 40 C/24 h						
L+T+BAC	Imp. H < RT ^2^	RRT 0.43—0.14%; RRT 0.47—0.15%	0.29%	97.8%	0.274	98.6%
L+BAC	Imp. H < RT ^2^	<RT ^2^	NA	97.3%	0.231	97.8%
Thermal degradation 80 C/24 h						
L+T+BAC	Imp. H—0.83%	<RT ^2^	0.83%	98.9%	0.259	100.2%
L+BAC	Imp. H—1.39%	<RT ^2^	1.39%	97.4%	0.275	99.3%

^1^—in relation to the declared assay of latanoprost in the medicinal product 50 µg/mL; ^2^—RT (Reporting Threshold) = 0.10%, ^3^—PA/PT—purity test parameters in Empower system PA—purity angle, PT—Purity threshold, PA/PT should be ≤ 1.0, *—product contained latanoprost with BAC was subjected to acid hydrolysis for 2 h, **—product contained latanoprost and timolol with BAC was subjected to acid hydrolysis for 2 h.

**Table 4:** Measuring system precision (Agilent system).

Inj. No.	Latanoprost ~50 µg/mL		Latanoprost ~0.5 µg/mL		Imp. H ~0.50 µg/mL		Imp. F ~0.88 µg/mL		Imp. E ~0.14 µg/mL	
	Peak Area [µS × s]	RT [min]	Peak Area [µS × s]	RT [min]	Peak Area [µS × s]	RT [min]	Peak Area [µS × s]	RT [min]	Peak Area [µS × s]	RT [min]
1	11,615,872	68.06	125,574	68.38	119,610	36.17	224,886	71.41	31,819	82.27
2	11,625,870	67.96	124,860	68.01	120,420	36.19	223,772	71.51	34,026	82.33
3	11,586,447	67.88	124,486	68.26	120,851	36.35	224,028	71.60	32,604	82.57
4	11,586,125	67.88	124,219	68.01	119,817	36.21	224,399	71.32	35,571	82.13
5	11,559,556	67.83	122,304	67.87	119,608	36.21	227,636	71.32	33,540	82.17
6	11,549,791	67.81	123,126	67.99	119,723	36.21	227,489	71.42	35,167	82.30
mean	11,587,277	67.90	124,095	68.09	120,005	36.22	225,368	71.43	33,788	82.30
SD	29,942	0.09	1191	0.19	513	0.06	1741	0.11	1448	0.16
RSD%	0.26	0.14	0.96	0.28	0.43	0.18	0.77	0.15	4.28	0.19

**Table 5:** Linearity, accuracy, and precision test results for latanoprost and latanoprost-related substances in the products latanoprost and timolol with BAC.

Performance Characteristic	Latanoprost Assay	Latanoprost Unknown Impurities	Imp. E	Imp. F	Imp. H
Range [µg/mL]	40–60 µg/mL	0.05–2.77 µg/mL	0.05–0.41 µg/mL	0.05–2.79 µg/mL	0.05–1.78 µg/mL
Accuracy					
Recovery 1	conc. ~40 µg/mL 99.0% (RSD_n=3_—1.0%)	conc. ~0.05 µg/mL 101.6% (RSD_n=3_—2.4%)	conc. ~0.08 µg/mL 102,0% (RSD_n=3_—2.9%)	conc. ~0.1 µg/mL 95.0 (RSD_n=3_—3.0%)	conc. ~0.08 µg/mL 91.7% (RSD_n=3_—0.6%)
Recovery 2	conc. ~50 µg/mL 99.8% (RSDn_=3_—0.5%)	conc. ~0.5 µg/mL 92.4% (RSD_n=3_—1.4%)	conc. ~0.3 µg/mL 97.3% (RSD_n=3_—5.8%)	conc. ~0.4 µg/mL 99.4% (RSD_n=3_—2.1%)	conc. ~0.8 µg/mL 92.8% (RSD_n=3_—0.6%)
Recovery 3	conc. ~60 µg/mL 99.3% (RSDn_=3_—0.1%)	conc. ~2.8 µg/mL 95.6% (RSD_n=3_—1.1%)	conc. ~0.4 µg/mL 96.9% (RSD_n=3_—1.4%)	conc. ~2 µg/mL 103.5% (RSD_n=3_—1.6%)	conc. ~1.5 µg/mL 93.6% (RSD_n=3_—1.4%)
Mean (R ± ΔR)	99.4% ± 0.5% *	96.5% ± 3.1% *	98.8% ± 3.1% *	99.3 ± 3.2% *	92.71 ± 0.9% *
SD	0.6% (*n* = 9)	4.1 (*n* = 9)	4.1 (*n* = 9)	4.2 (*n* = 9)	1.1 (*n* = 9)
RSD%	0.6% (*n* = 9)	4.2 (*n* = 9)	4.1 (*n* = 9)	4.2 (*n* = 9)	1.2 (*n* = 9)
Linearity					
Regression equation	Y = 239,779x − 75,565	Y = 234,862x − 178	Y = 242,443x + 1122	Y = 266,580x − 4835	Y = 237,817x − 630
Slope (b ± Δb)	239,779 ± 7437	234,862 ± 1926	242,443 ± 11246	266,580 ± 2623	237,817 ± 1552
Sb	3443	891	5206	1214	906
Intercept (a ± Δa)	−75,565 ± 385,507	−178 ± 2906	1122 ± 2779	−4835 ± 2812	−630 ± 1552
Sa	178,475	1345	1287	1302	719
Sxy	90,601	3689	2294	3560	1896
Correlation coefficient	0.999	0.999	0.997	0.999	0.999
*p* value **	4.10 × 10^−18^	1.27 × 10^−25^	7.53 × 10^−16^	1.37 × 10^−24^	1.34 × 10^−25^
Precision					
Repeatability	X_1(mean n=6)_ 95.65%	X_1(n=6)_ 0.20% (0.10 µg/mL)	X_1(n=6)_ 0.26% (0.13 µg/mL)	X_1(n=6)_ 1.89% (0.94 µg/mL)	X_1(n=6)_ 1.00% (0.50 µg/mL)
SD *n* = 6	0.47%	0.01% (0.00 µg/mL)	0.01% (0.01 µg/mL)	0.01% (0.01 µg/mL)	0.01% (0.00 µg/mL)
RSD	0.49%	2.81%	5.19%	0.64%	0.51%
Intermediate precision	X_2(mean n=6)_ 95.80%	X_2(n=6)_ 0.20% (0.10 µg/mL)	X_2(n=6)_ 0.29% (0.15 µg/mL)	X_2(n=6)_ 1.79% (0.89 µg/mL)	X_2(n=6)_ 0.97% (0.49 µg/mL)
SD *n* = 6	0.83%	0.01% (0.00 µg/mL)	0.01% (0.01 µg/mL)	0.01% (0.01 µg/mL)	0.00%
RSD *n* = 6	0.87%	3.16%	2.58%	0.74%	0.42%
Mean_n=12_ ± ΔX	X_(n=12)_ 95.73 ± 0.41%	X_(n=12)_ 0.20% (0.10 µg/mL)	X_(n=12)_ 0.28% (0.14 µg/mL)	X_(n=12)_ 1.84% (0.92 µg/mL)	X_(n=12)_ 0.99% (0.50 µg/mL)
$\left|X1-X2\right|$	0.15%	0.00% (0.00 µg/mL)	0.03% (0.02 µg/mL)	0.10% (0.05 µg/mL)	0.03% (0.01 µg/mL)
SD *n* = 12	0.65%	0.01% (0.00 µg/mL)	0.02% (0.01 µg/mL)	0.05% (0.03 µg/mL)	0.02% (0.01 µg/mL)
RSD *n* = 12	0.68%	3.15%	6.54%	2.87%	1.73%

* (T-student, *p* = 0.05, k = n-1 = 8), ** (F-statistics, *p* = 0.05, *n* = 15).

**Table 6:** Linearity, accuracy, and precision test results for latanoprost and latanoprost-related substances in the product latanoprost with BAC.

Performance Characteristic	Latanoprost Assay	Latanoprost Unknown Impurities	Imp. E	Imp. F	Imp. H
Range	40–60 µg/mL	0.05–2.77 µg/mL	0.05–0.41 µg/mL	0.05–2.79 µg/mL	0.05–1.78 µg/mL
**Accuracy**					
Recovery 1	conc. ~40 µg/mL 98.8% (RSD_n=3_—0.7%)	conc. ~0.05 µg/mL 93.2% (RSD_n=3_—2.5%)	conc. ~0.05 µg/mL 97.4% (RSD_n=3_—5.0%)	conc. ~0.05 µg/mL 98.1% (RSD_n=3_—1.9%)	conc. ~0.05 µg/mL 96.7% (RSD_n=3_—1.2%)
Recovery 2	conc. ~50 µg/mL 99.0% (RSD_n=3_—0.3%)	conc. ~1 µg/mL 93.7% (RSD_n=3_—1.7%)	conc. ~0.3 µg/mL 107.9% (RSD_n=3_—2.6%)	conc. ~2 µg/mL 103.6% (RSD_n=3_—0.4%)	conc. ~0.8 µg/mL 95.2% (RSD_n=3_—2.9%)
Recovery 3	conc. ~60 µg/mL 98.3% (RSD_n=3_—0.1%)	conc. ~2.8 µg/mL 96.8% (RSD_n=3_—0.9%)	conc. ~0.4 µg/mL 105.9% (RSD_n=3_—3.6%)	conc. ~2.9 µg/mL 105.1% (RSD_n=3_—0.7%)	conc. ~1.5 µg/mL 95.6% (RSD_n=3_—2.5%)
Mean (R ± ΔR)	98.7% ± 0.4% *	94.6% ± 1.7% *	103.7% ± 4.5% *	102.3% ± 2.6% *	95.8% ± 1.6% *
SD	0.5% (*n* = 9)	2.3 (*n* = 9)	5.9 (*n* = 9)	3.4 (*n* = 9)	2.0 (*n* = 9)
RSD%	0.5% (*n* = 9)	2.4 (*n* = 9)	5.7 (*n* = 9)	3.3 (*n* = 9)	2.1 (*n* = 9)
**Linearity**					
Regression equation	y = 231,609x + 48,800	y = 261,851x − 4725	y = 278,817x − 1220	y = 265,770x − 1769	y = 240,793x − 134
Slope (b ± Δb)	231,609 ± 1515	261,851 ± 1926	278,817 ± 3794	265,770 ± 2270	240,793 ± 6485
Sb	701	1796	1757	1051	3002
Intercept (a ± Δa)	48,800 ± 80,231	−4725 ± 5988	−1220 ± 1045	−1769 ± 4222	−134 ± 6479
Sa	37,144	2772	484	1955	2999
Sxy	45,855	6686	814	4051	6026
Correlation coefficient (r)	0.999	0.999	0.999	0.999	0.999
*p* value **	6.78 × 10^−27^	2.69 × 10^−22^	9.71 × 10^−23^	2.27 × 10^−25^	6.80 × 10^−19^
**Precision**					
Repeatability	X_1(mean n=6)_ 95.65%	<LOD (0.05%)	X_1(n=6)_ 0.23% (0.11 µg/mL)	X_1(n=6)_ 2.52% (1.26 µg/mL)	X_1(n=6)_ 0.55% (0.28 µg/mL)
SD *n* = 6	0.47%	NA	0.01% (0.00 µg/mL)	0.01% (0.00 µg/mL)	0.00% (0.00 µg/mL)
RSD	0.49%	NA	3.30%	0.21%	0.00%
Intermediate precision	X_2(mean n=6)_ 95.80%	<LOD (0.05%)	X_2(n=6)_ 0.32% (0.16 µg/mL)	X_2 (n=6)_ 2.57% (1.28 µg/mL)	X_2(n=6)_ 0.65% (0.33 µg/mL)
SD *n* = 6	0.83%	NA	0.02% (0.01 µg/mL)	0.05% (0.02 µg/mL)	0.01% (0.00 µg/mL)
RSD *n* = 6	0.87%	NA	5.59%	1.84%	0.92%
Mean *n* = 12	X_(n=12)_ 95.73 ± 0.41%	NA	X_(n=12)_ 0.27% (0.14 µg/mL)	X_(n=12)_ 2.54% (1.27 µg/mL)	X_(n=12)_ 0.60% (0.30 µg/mL)
$\left|X1-X2\right|$	0.15%	NA	0.09% (0.05 µg/mL)	0.05% (0.03 µg/mL)	0.10% (0.05 µg/mL)
SD *n* = 12	0.65%	NA	0.05% (0.02 µg/mL)	0.04% (0.02 µg/mL)	0.05% (0.03 µg/mL)
RSD *n* = 12	0.68%	NA	18.10%	1.63%	8.73%

* (T-student, *p* = 0.05,k = n−1 = 8), ** (F-statistics, *p* = 0.05, *n* = 15).

**Table 8:** Limits of detection and quantification calculated from regression curves for latanoprost and impurities F, H, and E tested in both mono and combo products.

Tested Product Parameter	Latanoprost	Imp. F	Imp. H	Imp. E
L+BAC				
Slope (b)	261,851	265,770	240,793	278,817
Sy	2772	1955	2999	484
LOQ [µg/mL]	0.11	0.07	0.12	0.02
LOD [µg/mL]	0.03	0.02	0.04	0.01
L+T+BAC				
Slope (b)	234,862	266,580	237,817	242,443
Sy	1345	1302	719	1287
LOQ [µg/mL]	0.06	0.05	0.03	0.05
LOD [µg/mL]	0.02	0.01	0.01	0.02

**Table 9:** Experimentally determined limits of detection and quantification.

Limit	Latanoprost Concentration µg/mL (%) ^1^	S/N ^2^	RSD (*n* = 6)
LOD	0.025 (0.05%)	19	6.0%
LOQ	0.035 (0.07%)	27	2.4%

^1^—in relation to declared latanoprost content 50 µg/mL, ^2^ S/N—signal to noise ratio calculated by Empower Software acc. Ph. Eur.

**Table 10:** Method robustness: SST results with small changes in analysis conditions.

Analysis Conditions	SST Parameters		
	Reference Solution of Latanoprost 1.0%		SST Solution
	RSD(%) Latanoprost Peak Area (*n* = 5)	As ***	R_s_ ****
Acc. TM *	1.25	1.1	2.1
column temperature: −2 °C **	1.97	1.1	2.1
column temperature: +2 °C **	2.01	1.1	2.1
Acc. TM *, chromatographic system set 1	1.73	1.1	2.0
Acc. TM *, chromatographic system set 2	1.58	1.1	1.9
Acc. TM *	1.46	1.1	2.2
−2% of acetonitrile in mobile phase **	0.36	1.1	2.0
+2% of acetonitrile in mobile phase **	1.52	1.1	2.1
SST Acceptance criteria:	RSD(%) ≤ 5.0	0.8 ≤ As ≤ 1.5	$R_{s}$ ≥ 1.8

*—Acc. TM—according to parameters set in test method, **—in relation to settings in tested method, ***—As—symmetry factor of latanoprost peak, **** Rs—resolution between peak of impurity F and latanoprost.

**Table 11:** Method robustness: Xalatan product results analyzed with small changes in test conditions.

Analysis Conditions	Results			
	Related Substances			Assay
	Impurity H	Impurity F	Impurity E	latanoprost
Acc. TM *	0.55% ***	2.47% ***	0.22% ***	92.7% ***
column temperature: −2 °C **	0.52% ***	2.28% ***	0.19% ***	91.8% ***
column temperature: +2 °C **	0.54% ***	2.45% ***	0.18% ***	92.8% ***
	RSD% 2.85	RSD% 4.35	RSD% 10.58	RSD% 0.60
Acc. TM *, chromatographic set 1	0.55% ***	2.52% ***	0.23% ***	92.7% ***
Acc. TM *, chromatographic set 2	0.65% ***	2.60% ***	0.28% ***	93.8% ***
	RSD% 11.79	RSD% 2.21	RSD% 13.86	RSD% 0.83
Acc. TM *	0.59% ***	2.68% ***	0.24% ***	93.8% ***
−2% of acetonitrile in mobile phase **	0.66% ***	2.43% ***	0.19% ***	93.9% ***
+2% of acetonitrile in mobile phase **	0.64% ***	2.50% ***	0.30% ***	93.7% ***
	RSD% 5.72	RSD% 5.08	RSD% 22.63	RSD% 0.11

*—Acc. TM—according to parameters set in test method, **—in relation to settings in tested method, ***—in relation to declared latanoprost content 50 µg/mL.

**Table 12:** Assay results and related substances obtained for Xalatan and latanoprost products: 50 µg/mL (benzalkonium chloride-free).

	Xalatan Batch 1	Xalatan Batch 2	Latanoprost Formulation BAC Free Batch 1	Latanoprost, Formulation BAC Free Batch 2
Latanoprost assay	92.4%	92.8%	93.6%	95.3%
Impurity F	2.27%	2.93%	<RT *	<RT *
Impurity E	0.14%	0.14%	<RT *	<RT *
Impurity H	1.48%	0.89%	1.02%	1.05%
Impurity D	<RT *	<RT *	<RT *	<RT *
TPPO	<RT *	<RT *	<RT *	<RT *
Latanoprost enantiomer	<RT *	<RT *	<RT *	<RT *
15-ketolatanoprost	<RT *	<RT *	<RT *	<RT *
Total	3.9%	4.0%	1.8%	2.1%

*—reporting threshold (RT) 0.1% in relation to 50 µg/mL.

**Table 13:** Assay results and related substances obtained for Xalacom and latanoprost products with timolol (50 µg + 5 mg)/mL (benzalkonium chloride-free).

	Xalacom Batch 1	Xalacom Batch 2	Latanoprost, Timolol Formulation BAC Free Batch 1	Latanoprost, Timolol Formulation BAC Free Batch 2
Latanoprost assay	96.4%	101.8%	97.6%	99.2%
Impurity F	2.0%	2.7%	0.10%	0.10%
Impurity E	0.10%	<0.10%	<RT *	<RT *
Impurity H	<0.10%	0.35%	1.2%	1.2%
Impurity D	<RT *	<RT *	<RT *	<RT *
TPPO	<RT *	<RT *	<RT *	<RT *
Latanoprost enantiomer	<RT *	<RT *	<RT *	<RT *
15-ketolatanoprost	<RT *	<RT *	<RT *	<RT *
Total	2.1%	3.4%	1.5%	1.9%

*—reporting threshold (RT) 0.1% in relation to 50 µg/mL.

**Table 14:** The mobile phase gradient program.

Time Point (min)	Flow (mL/min)	Phase A (% *v*/*v*)	Phase B (% *v*/*v*)
0	0.5	100	0
18.0	0.5	100	0
30.0	0.5	85	15
60.0	0.5	85	15
75.0	0.4	88	12
100.0	0.5	88	12
115.0	0.5	88	12
116.0	1.0	50	50
127.0	0.5	50	50
128.0	1.0	50	50
140.0	0.5	100	0

## Data Availability

The original comments presented in this study are included in the article; further inquiries can be directed to the corresponding author. Due to trade secrets, the authors are not permitted to share the data.

## Supplementary Materials

The following supporting information can be downloaded at: https://www.mdpi.com/article/10.3390/ph18050619/s1, Figure S1: Degradation L+BAC 80C_24h, Figure S2: Degradation L+BAC_1h UV, Figure S3: Degradation L+BAC_NaOH_4h, Figure S4: L+T+BAC before degradation, Figure S5: Degradation L+T+BAC 40C_24h, Figure S6: Degradation L+T+BAC_1hUV.
