# Supplementary material for: Developing a Chromatographic Method for Quantifying Latanoprost and Related Substances in Glaucoma Treatments
Source: Pharmaceuticals (Basel). 2025 Apr 24;18(5):619. doi: 10.3390/ph18050619 (PMC12114650; doi:10.3390/ph18050619)
Supplement: Supplementary file 1 [file pharmaceuticals-18-00619-s001.zip › S1 Degradation L+BAC 80C_24h.pdf]

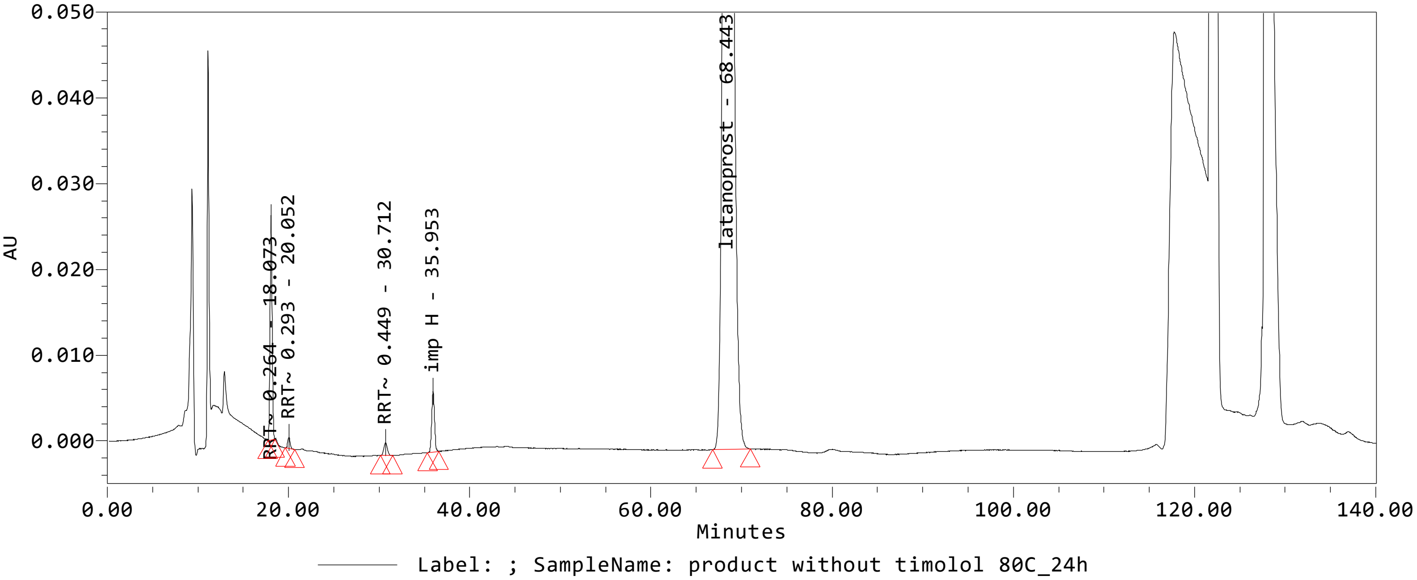

SampleName: product without timolol 80C_24h

	SampleName	Name	RT	RRT	Dilution	Area	X_imp
1	product without timolol 80C_24h	RRT~ 0.264	18.1	0.26	1.0000	425364	3.48
2	product without timolol 80C_24h	RRT~ 0.293	20.1	0.29	1.0000	25769	0.21
3	product without timolol 80C_24h	RRT~ 0.449	30.7	0.45	1.0000	41017	0.34
4	product without timolol 80C_24h	imp H	36.0	0.53	1.0000	152382	1.38
