# Supplementary material for: Developing a Chromatographic Method for Quantifying Latanoprost and Related Substances in Glaucoma Treatments
Source: Pharmaceuticals (Basel). 2025 Apr 24;18(5):619. doi: 10.3390/ph18050619 (PMC12114650; doi:10.3390/ph18050619)
Supplement: Supplementary file 1 [file pharmaceuticals-18-00619-s001.zip › S2 Degradation L+BAC_1h UV.pdf]

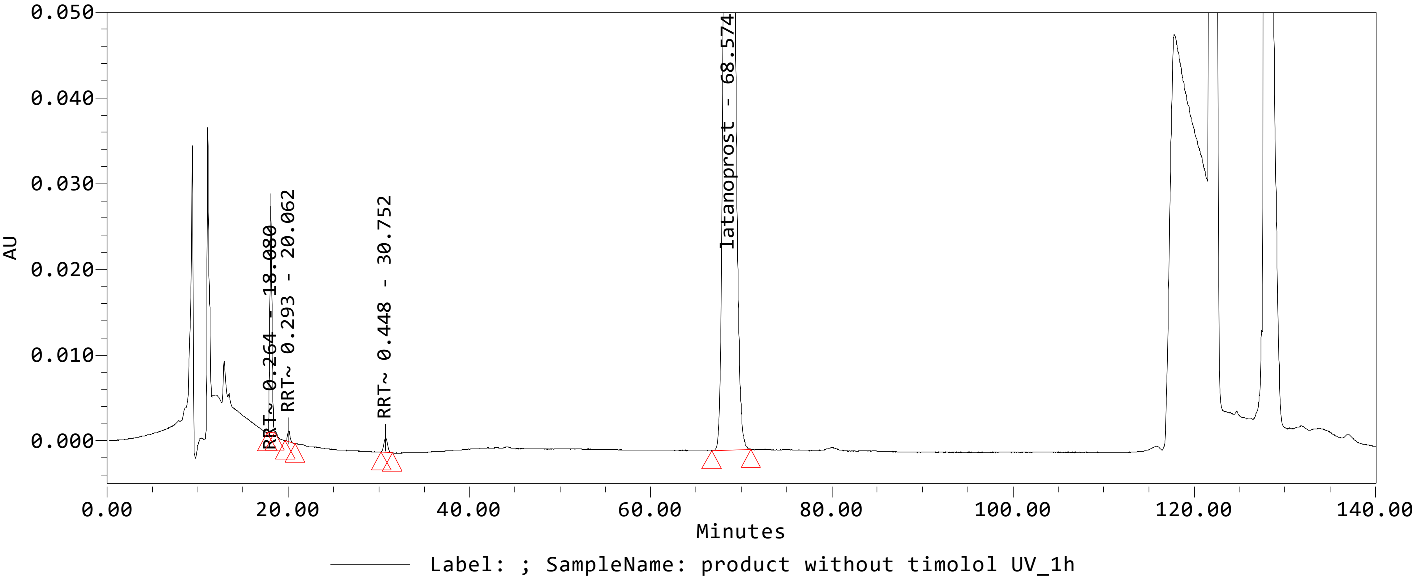

SampleName: product without timolol UV_1h

	SampleName	Name	RT	RRT	Dilution	Area	X_imp
1	product without timolol UV_1h	RRT~ 0.264	18.1	0.26	1.0000	429603	3.52
2	product without timolol UV_1h	RRT~ 0.293	20.1	0.29	1.0000	25840	0.21
3	product without timolol UV_1h	RRT~ 0.448	30.8	0.45	1.0000	47805	0.39
