# Supplementary material for: Developing a Chromatographic Method for Quantifying Latanoprost and Related Substances in Glaucoma Treatments
Source: Pharmaceuticals (Basel). 2025 Apr 24;18(5):619. doi: 10.3390/ph18050619 (PMC12114650; doi:10.3390/ph18050619)
Supplement: Supplementary file 1 [file pharmaceuticals-18-00619-s001.zip › S3 Degradation L+BAC_NaOH_4h.pdf]

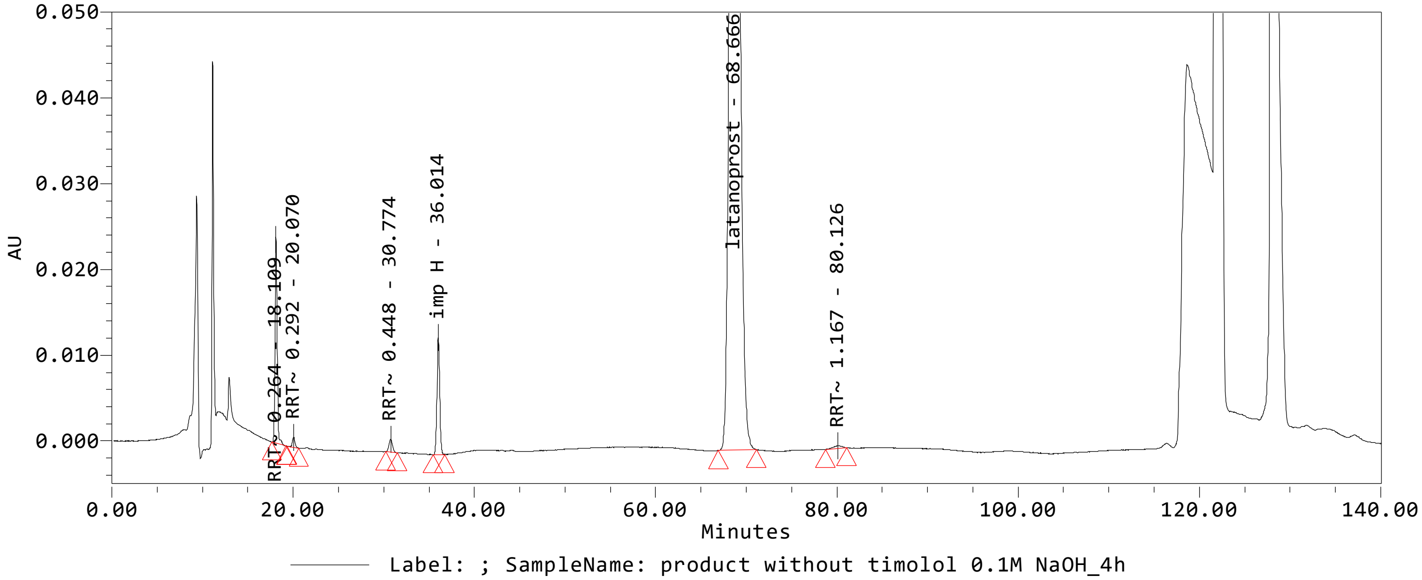

SampleName: product without timolol 0.1M NaOH_4h

	SampleName	Name	RT	RRT	Dilution	Area	X_imp
1	product without timolol 0.1M NaOH_4h	RRT~ 0.264	18.1	0.26	1.2500	406234	4.16
2	product without timolol 0.1M NaOH_4h	RRT~ 0.292	20.1	0.29	1.2500	23504	0.24
3	product without timolol 0.1M NaOH_4h	RRT~ 0.448	30.8	0.45	1.2500	41337	0.42
4	product without timolol 0.1M NaOH_4h	imp H	36.0	0.52	1.2500	291601	3.31
5	product without timolol 0.1M NaOH_4h	RRT~ 1.167	80.1	1.17	1.2500	22613	0.23
