# Supplementary material for: Developing a Chromatographic Method for Quantifying Latanoprost and Related Substances in Glaucoma Treatments
Source: Pharmaceuticals (Basel). 2025 Apr 24;18(5):619. doi: 10.3390/ph18050619 (PMC12114650; doi:10.3390/ph18050619)
Supplement: Supplementary file 1 [file pharmaceuticals-18-00619-s001.zip › S4 L+T+BAC before degradation.pdf]

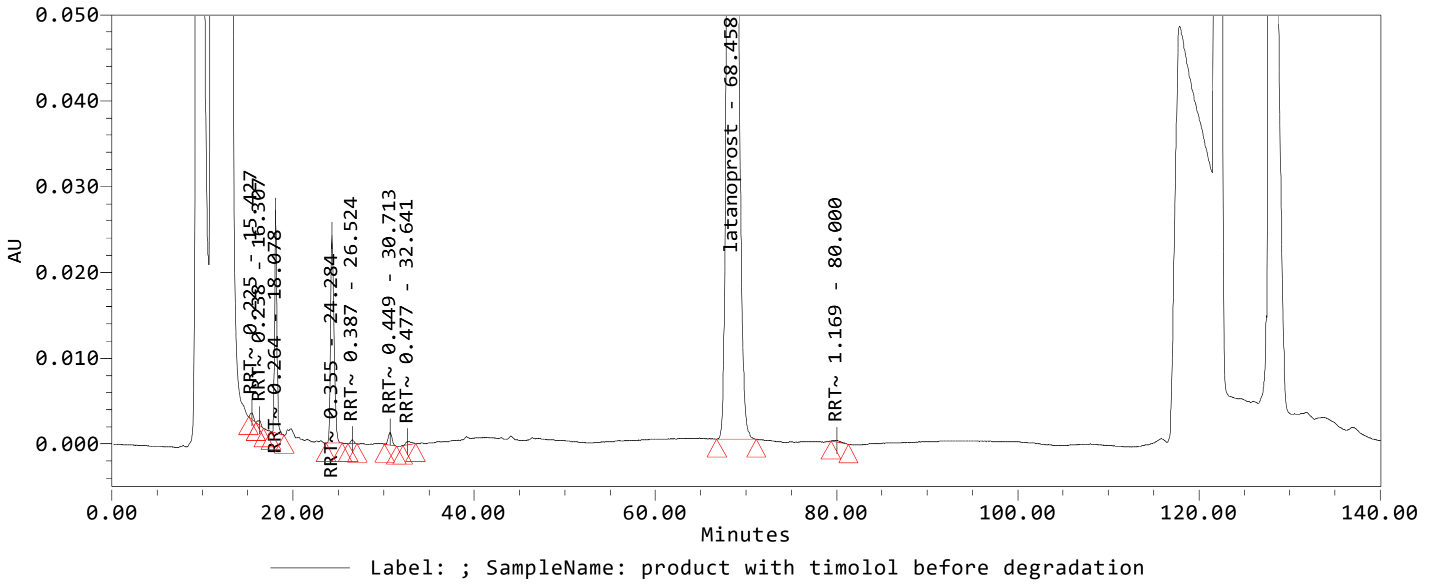

SampleName: product with timolol before degradation

	SampleName	Name	RT	RRT	Dilution	Area	X_imp
1	product with timolol before degradation	RRT~ 0.225	15.4	0.23	1.0000	17777	0.15
2	product with timolol before degradation	RRT~ 0.238	16.3	0.24	1.0000	14036	0.11
3	product with timolol before degradation	RRT~ 0.264	18.1	0.26	1.0000	446865	3.66
4	product with timolol before degradation	RRT~ 0.355	24.3	0.35	1.0000	643768	5.27
5	product with timolol before degradation	RRT~ 0.387	26.5	0.39	1.0000	12051	0.10
6	product with timolol before degradation	RRT~ 0.449	30.7	0.45	1.0000	43523	0.36
7	product with timolol before degradation	RRT~ 0.477	32.6	0.48	1.0000	16606	0.14
8	product with timolol before degradation	RRT~ 1.169	80.0	1.17	1.0000	12491	0.10
