# Supplementary material for: Developing a Chromatographic Method for Quantifying Latanoprost and Related Substances in Glaucoma Treatments
Source: Pharmaceuticals (Basel). 2025 Apr 24;18(5):619. doi: 10.3390/ph18050619 (PMC12114650; doi:10.3390/ph18050619)
Supplement: Supplementary file 1 [file pharmaceuticals-18-00619-s001.zip › S5 Degradation L+T+BAC 40C_24h.pdf]

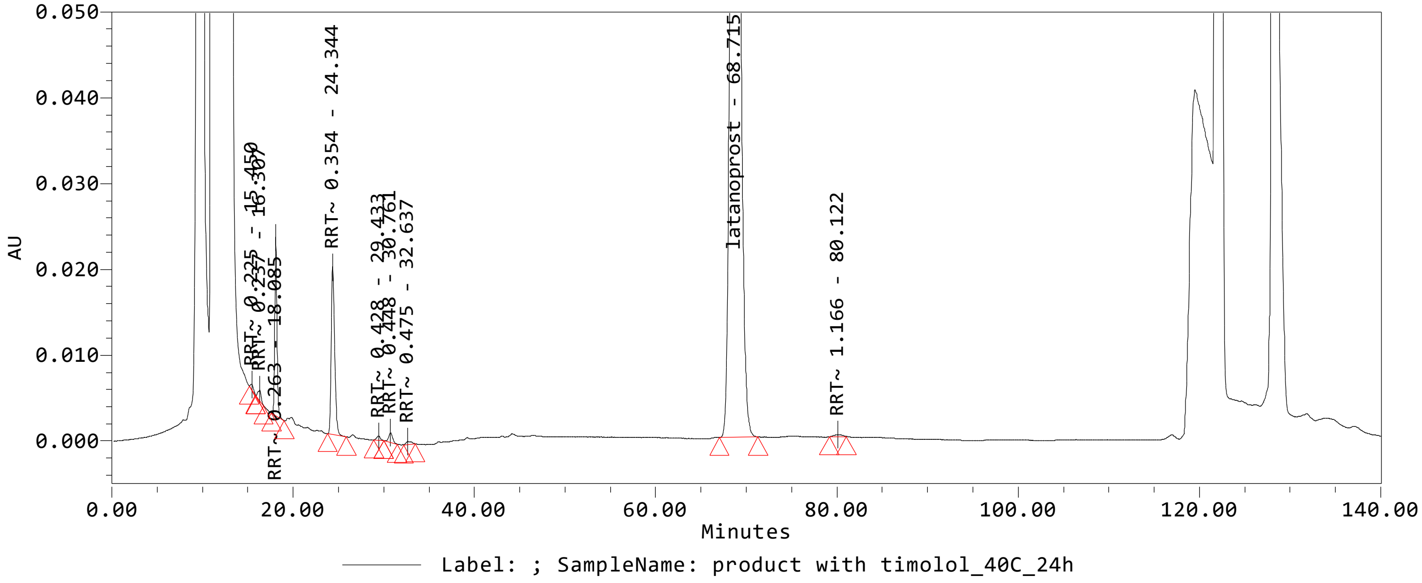

SampleName: product with timolol_40C_24h

	SampleName	Name	RT	RRT	Dilution	Area	X_imp
1	product with timolol_40C_24h	RRT~ 0.225	15.4	0.22	1.0000	13777	0.11
2	product with timolol_40C_24h	RRT~ 0.237	16.3	0.24	1.0000	29252	0.24
3	product with timolol_40C_24h	RRT~ 0.263	18.1	0.26	1.0000	356245	2.92
4	product with timolol_40C_24h	RRT~ 0.354	24.3	0.35	1.0000	521530	4.27
5	product with timolol_40C_24h	RRT~ 0.428	29.4	0.43	1.0000	13671	0.11
6	product with timolol_40C_24h	RRT~ 0.448	30.8	0.45	1.0000	33251	0.27
7	product with timolol_40C_24h	RRT~ 0.475	32.6	0.47	1.0000	14906	0.12
8	product with timolol_40C_24h	RRT~ 1.166	80.1	1.17	1.0000	14616	0.12
