# Supplementary material for: Developing a Chromatographic Method for Quantifying Latanoprost and Related Substances in Glaucoma Treatments
Source: Pharmaceuticals (Basel). 2025 Apr 24;18(5):619. doi: 10.3390/ph18050619 (PMC12114650; doi:10.3390/ph18050619)
Supplement: Supplementary file 1 [file pharmaceuticals-18-00619-s001.zip › S6 Degradation L+T+BAC_1hUV.pdf]

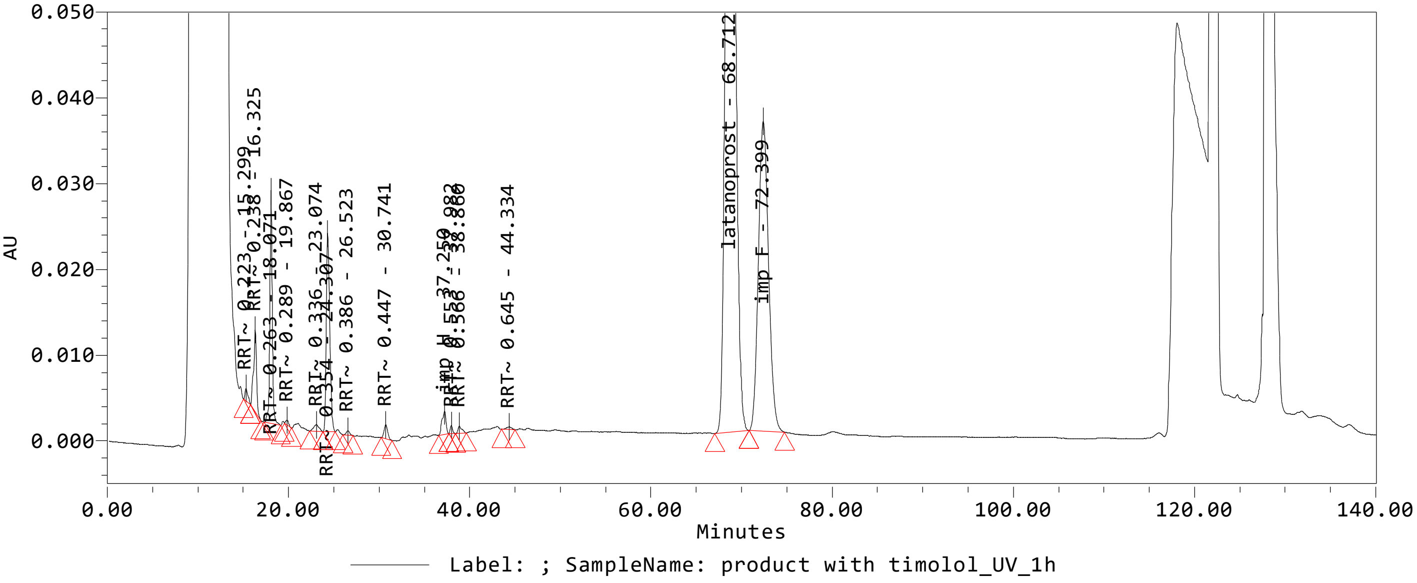


SampleName: product with timolol_UV_1h

| | SampleName | Name | RT | RRT | Dilution | Area | X_imp |
|---|---|---|---|---|---|---|---|
| 1 | product with timolol_UV_1h | RRT~ 0.223 | 15.3 | 0.22 | 1.0000 | 33800 | 0.28 |
| 2 | product with timolol_UV_1h | RRT~ 0.238 | 16.3 | 0.24 | 1.0000 | 215158 | 1.76 |
| 3 | product with timolol_UV_1h | RRT~ 0.263 | 18.1 | 0.26 | 1.0000 | 513262 | 4.20 |
| 4 | product with timolol_UV_1h | RRT~ 0.289 | 19.9 | 0.29 | 1.0000 | 13014 | 0.11 |
| 5 | product with timolol_UV_1h | RRT~ 0.336 | 23.1 | 0.34 | 1.0000 | 31918 | 0.26 |
| 6 | product with timolol_UV_1h | RRT~ 0.354 | 24.3 | 0.35 | 1.0000 | 617637 | 5.06 |
| 7 | product with timolol_UV_1h | RRT~ 0.386 | 26.5 | 0.39 | 1.0000 | 13480 | 0.11 |
| 8 | product with timolol_UV_1h | RRT~ 0.447 | 30.7 | 0.45 | 1.0000 | 45248 | 0.37 |
| 9 | product with timolol_UV_1h | imp H | 37.3 | 0.54 | 1.0000 | 82562 | 0.75 |
| 10 | product with timolol_UV_1h | RRT~ 0.553 | 38.0 | 0.55 | 1.0000 | 17150 | 0.14 |
| 11 | product with timolol_UV_1h | RRT~ 0.566 | 38.9 | 0.57 | 1.0000 | 25300 | 0.21 |
| 12 | product with timolol_UV_1h | RRT~ 0.645 | 44.3 | 0.65 | 1.0000 | 12623 | 0.10 |
| 13 | product with timolol_UV_1h | imp F | 72.4 | 1.05 | 1.0000 | 2797546 | 22.90 |
